# Chronic Pain: A Complex Condition With a Multi-Tangential Approach

**DOI:** 10.7759/cureus.19850

**Published:** 2021-11-23

**Authors:** Iljena Kela, Chandra L Kakarala, Mohammad Hassan, Rishab Belavadi, Sri Vallabh Reddy Gudigopuram, Ciri C Raguthu, Harini Gajjela, Ibrahim Sange

**Affiliations:** 1 Family Medicine, Jagiellonian University Medical College, Krakow, POL; 2 Internal Medicine, Jawaharlal Institute of Post-Graduate Medical Education and Research (JIPMER), Puducherry, IND; 3 Internal Medicine, Mohiuddin Islamic Medical College, Mirpur, PAK; 4 Surgery, Jawaharlal Institute of Post-Graduate Medical Education and Research (JIPMER), Puducherry, IND; 5 Research, Our Lady of Fatima University College of Medicine, Manila, PHL; 6 Research, Tianjin Medical University, Tianjin, CHN; 7 Research, Our Lady of Fatima University College of Medicine Valenzuela, Metro Manila, PHL; 8 Research, K. J. Somaiya Medical College, Mumbai, IND

**Keywords:** nonpharmacological treatment of chronic pain, pharmacological management of chronic pain, neuropathic pain treatment, chronic pain, nociceptive pain, neuropathic pain, chronic pain management, chronic pain treatment

## Abstract

Chronic pain is known as ongoing pain that lasts longer than three months with increasing healing time. It is approximated that 20% of adults of different sexes, races, and socioeconomic backgrounds fall victim to chronic pain. It is a result of several factors and can have lifelong effects. Pain is a complex matter to measure; therefore, the physician needs to understand the patient’s health state to create a management plan tending to each issue adequately. There are many complications of such pain, and it can interfere terribly with an individual’s quality of life. This article has reviewed the complex pathogenesis of chronic pain and the spectrum of non-pharmacologic modalities and pharmacological treatment options. It has also explored the efficacy of certain drugs and underlined the importance of nonpharmacological options such as physical exercise, cognitive therapy, and physical modalities to treat chronic pain and all the conditions that accompany this disorder.

## Introduction and background

By definition, recurring pain lasting longer than three months with a relatively amplified duration of the healing time is known as chronic pain [1]. Chronic pain is diagnosed within 10% of individuals globally, and it is approximated that 20% of adults suffer from pain worldwide [2]. Chronic pain can result from several factors such as trauma (psychological and physical), a chronic disease, especially autoimmune conditions, or infections [3]. From the genetic perspective, children with a family history of chronic pain in either parent tend to have a lower threshold for pain and are associated with adverse health and physical outcomes in the long run [4]. Chronic pain can be primarily divided into two main types - neuropathic and nociceptive. There is a mixed type of pain known as nociplastic. Neuropathic pain is characterized by damaged nerve endings sending spontaneous signals to the brain and spinal cord (sciatica, peripheral neuropathy) [5]. Nociceptive pain occurs predominantly when the activated nociceptors (pain receptors) malfunction due to trauma and send abnormal signals to the brain, which are perceived even after the injury has healed [6]. The differences are summarized in Table 1.

**Table 1:** Differences between neuropathic and nociceptive pain

	Neuropathic pain	Nociceptive pain
Cause	Nerve damage	Inflammation
Severity	Increases over time	Decreases over time
Duration	Chronic	Chronic, but can resolve over time
Examples	Trigeminal Neuralgia	Rheumatoid Arthritis

According to the International Classification of Disease of the World Health Organization, social, biological, and psychological factors contribute to chronic pain as it affects individuals of different sexes, races, socioeconomic backgrounds, and ages [1]. The incidence of chronic pain is shown to have a slight disposition towards African-American ethnicity and depicts a linear association with the age group of the patients [7]. A United Kingdom study showed that the prevalence increased with increasing age as 14.3% of individuals aged 18-25 years presented with chronic pain compared to 62% in the above 75 age group [8]. There are no typical chronic pain clinical presentations as pain can vary in location, precipitating factors, quality of pain, severity, etc. [5]. Pain is a subjective and individual matter and is therefore coherently challenging to measure. Therefore, obtaining history specific to neurologic, urologic, GI, gynecologic, and musculoskeletal-related disorders can be useful. The diagnostic investigation usually starts with a thorough history and physical examination, followed by diagnostic imaging or laboratory tests to establish the origin of the pain [9]. If there is no evidence of pathology, the physician may assume that the root of the pain can be due to psychological factors [9]. Imagining studies, diagnostic nerve blocks, and electrodiagnostic testing can aid in pain evaluation. A plain radiograph is used along with magnetic resonance imaging to determine the source of pain. However, most conventional imaging studies cannot identify the primary source of pain [10]. Sleep complaints, anxiety, and depression disorders, along with chronic pain, form a distress triad that demonstrates significant societal and personal burdens for the sufferers [11]. These are all complications of chronic pain and interfere with an individual's life and daily activities. The consequences of chronic pain hamper a person’s ability to carry out activities of daily living with subsequent harmful impacts on mental and physical health, which creates an environment for a chaotic management plan and a grim prognosis. This review article aims to underline the pathogenesis of chronic pain and explore the spectrum of pharmacological and nonpharmacological treatment options.

## Review

Pathogenesis of chronic pain

The choice of therapy is dependent on many factors, but primarily on the type of chronic pain, the patient is experiencing. There are three types of chronic pain: nociceptive, neuropathic, and nociplastic (mixed).

Neuropathic pain is due to damage or pathology of the somatosensory nervous system and disorganized pain modulation centrally or peripherally [12]. Ectopic activity in nerve fibers is induced by a peripheral nerve lesion like nerve trauma, autoimmune disorders, postherpetic neuralgia, etc. [13]. In the dorsal root, ganglions are located the cell bodies of the peripheral somatosensory neurons [13]. In the dorsal aspect of the spinal cord and the dorsal root ganglion, the innate immune cells respond at the lesion site [13]. Chemical mediators that control the activity of neurons in the area are released by the active microglia of the dorsal horn [13]. Glycine’s and g-aminobutyric acid’s (GABA) inhibitory effect is reduced by brain-derived neurotrophic factor [13]. The polysynaptic connection in the dorsal horn is opened due to disinhibition [13]. The increase of glutaminergic transmission causes excitotoxic cell death, reducing the number of inhibitory interneurons [13]. The reduction shifts descending pathways from the brainstem, causing an imbalance between excitation and inhibition [13].

Nociceptive pain is due to the triggering of receptors sensitive to noxious stimuli [13]. Intense or prolonged exposure to these stimuli causes increased responsiveness of nociceptive nerve fibers [14]. Peripheral sensitization requires s a shift in the activation threshold of nociceptors and overexpressed voltage-gated sodium channels [13]. This shift leads to increased firing of the action potential and transmitter release, where the somatosensory information is processed in the dorsal horn of the spinal cord [13].

Enhanced depolarization leads to the engagement of N-methyl-D-aspartate (NMDA) type glutamate receptors [13]. Neuropeptide and NMDA receptor activation produces an increase in intracellular calcium, causing signaling pathways and gene expression that promote a shift in the function of nociceptive circuits [15].

Nociplastic pain is the third category of pain. The mechanism is poorly understood but thought that increased central nervous system pain, altered pain modulation, and sensory processing play prominent roles [16]. There is no evidence of tissue damage nor disease or lesion causing the pain but, nociceptors are still activated [17].

The symptoms include multifocal pain that is more intense or widespread, and sleep disturbances, mood problems, and fatigue [16]. This type of pain will respond to different therapies with low responsiveness to anti-inflammatory drugs, surgery, injections, and opioids [16].

Nonpharmacological treatment

Patient education about their chronic condition is essential as they are more likely to have better outcomes in their treatment [18]. Modern chronic pain treatment does not result in complete elimination of pain but an improvement of about 30% reduction on average [19]. Some nonpharmacological treatments include physical therapy, psychological therapy, and other physical modalities. Additionally, physicians must discuss better sleep hygiene with all patients suffering from chronic pain as disturbances during sleep have been shown to increase pain perception, suffering and negatively impact prognosis [20].

Physical therapy includes specified exercises that aid in conditioning patients with chronic pain allowing for patients to become more mobile and feel secure when doing daily activities [21]. Physical therapy shows small to moderate effects on disability and pain and some benefits for depression, anxiety, and the quality of life [21]. In 2017, a systematic review of small, randomized trials including physical activity and exercise for chronic pain found that exercise can improve function and lower pain with minor adverse effects (Table 2) [22]. As per a study published in 2017, the American College of Physicians showed randomized trials of nine non-pharmacologic options versus waitlist, sham treatment, or usual care or of one nonpharmacologic choice versus the other [23]. The number of trials evaluating nonpharmacological therapies ranged from two (tai chi) to 121 (exercise) [23]. Studies found that tai chi and mindfulness-based stress reduction are effective for chronic low back pain [23]. Stretching has also been found to reduce chronic low back pain and improve mobility over some time [24]. The American College of Physicians developed specific guidelines based on studies performed until 2015 to present evidence and clinical recommendations on noninvasive treatment of low back pain [24]. The first recommendation is that for most patients with acute or subacute low back pain, clinicians should choose nonpharmacologic treatment with superficial heat, acupuncture, massage, or spinal manipulation [24]. Furthermore, clinicians and patients should choose a treatment plan for patients with chronic low back pain, including exercise, acupuncture, tai chi, yoga, spinal manipulation, or cognitive behavioral therapy (CBT) [24]. A randomized controlled trial comparing 228 patients completing yoga, stretching, or a self-care book for chronic low back pain, found that the 12-week outcomes for the 92 patients subjected to yoga classes were superior to those 45 patients using the self-care book (Table 2) [25]. Yoga was not more effective than stretching classes in improving symptoms and function due to chronic low back pain (Table 2) [25]. Furthermore, patients can also partake in aquatic exercises as patients with pain and knee, and hip osteoarthritis may prefer them (Table 2) [26]. In a randomized controlled clinical trial of aquatic exercise following databases up to 2015, compared to a control group of about 1190 participants with knee or hip osteoarthritis, aquatic exercise caused a slight, short-term improvement compared to control in pain and disability (Table 2) [26]. A combination of endurance, flexibility, active strengthening, and balance will improve musculoskeletal function [27].

A range of different psychological approaches for managing chronic pain is analyzed as per the latest literature (Table 2) [28]. Individuals suffering from chronic pain show an increased tendency to develop other psychiatric conditions like posttraumatic stress disorder and anxiety disorders [29]. CBT offers a biopsychosocial approach to chronic pain management by selecting environmental and social occurrences that modify pain reactions and targeting unstable cognitive and behavioral responses (Table 2) [30]. In a randomized trial of adult patients with widespread chronic pain, 8% of patients were assigned to usual care reported symptom improvement at six months, 35% were assigned to CBT via telephone, and 37% were assigned to a combination of exercise and CBT via telephone (Table 2) [30]. A meta-analysis of 59 randomized trials of CBT versus no treatment or other forms of behavioral therapy in patients with chronic pain found minor benefits from CBT for disability, distress, or pain (Table 2) [28]. Additionally, a study with 342 adults with chronic low back pain was assigned randomly to receive mindfulness-based stress reduction therapy (MBSRT), CBT, or usual care delivered in eight weekly two-hour groups (Table 2) [31]. Treatment with MBSRT and CBT resulted in higher improvement in back pain and function (Table 2) [31]. 

**Table 2:** Summary of the studies showing outcomes of various non-pharmacological treatment modalities CBT- Cognitive- behavioral therapy

References	Design	Population/ data source	Conclusion
Boriosovskava et al. 2017 [22]	Systematic review	Ovid MEDLINE (January 2008 til February 2016), Cochrane Central Register of Controlled Trials, Cochrane Database of Systematic Reviews, and reference lists.	Several nonpharmacologic therapies for chronic back pain are associated with short term, minor to moderate effects on pain
Sherman et al. 2011 [25]	Randomized control trial	228 adults with chronic low back pain took part in the study. Ninety-two patients were randomized to 12 weekly classes, 91 patients to conventional stretching exercises, and 45 to self-care books.	Self-care books were less effective than yoga. However, yoga was not significantly more effective than stretching classes in reducing symptoms and improving function due to chronic low back pain, with benefits lasting at least several months.
Bartels et al. 2016 [26]	Systematic review	Following databases searched up to April 28, 2015: the Cochrane Central Register of Controlled Trials (CENTRAL; the Cochrane Library Issue 1, 2014), MEDLINE (from 1949), EMBASE (from 1980), CINAHL (from 1982), PEDro (Physiotherapy Evidence Database), and Web of Science (from 1945). 1190 participants	Aquatic exercise can have small, short term, and clinically relevant effects on patient-reported disability, pain, and quality of life in people with hip and knee osteoarthritis
Williams et al. 2020 [28]	Systematic review	9401 with fibromyalgia, rheumatoid arthritis, chronic low back pain, or mixed chronic pain	CBT has minimal effects for reducing disability, distress, and pain in chronic pain.
McBeth et al. 2012 [30]	Randomized controlled trial	442 patients fulfilling American College of Rheumatology criteria	Telephone-delivered cognitive behavioral therapy is associated with statistically significant improvement in global patient assessment
Cherkin et al. 2016 [31]	Randomized controlled trial	342 adults aged 20 to 70 years old with chronic low back pain	Treatment with mindfulness-based stress reduction or CBT resulted in more significant improvement in functional limitations and back pain than usual care at weeks 26.

There are also physical modalities such as transcutaneous electrical nerve stimulation (TENS). Transcutaneous electrical nerve stimulation (TENS) decreases central excitability and activates the central inhibition pathways [32]. Some current study suggests that TENS has relieved fibromyalgia symptoms for a short term while the stimulator is active [32].

**Figure 1:**
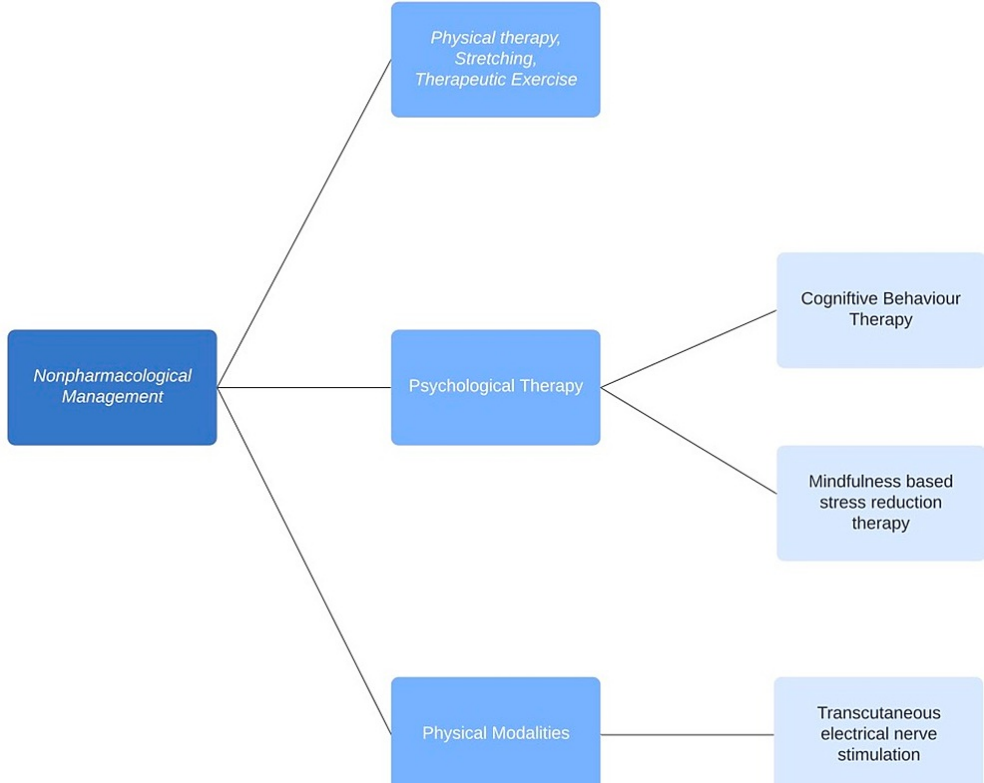
Summary of the different therapies in the nonpharmacological treatment of chronic pain

Continuous data displays the effectiveness of psychological therapies, spinal manipulation, massage, and acupuncture for chronic low back pain (Table 2) [23]. However, the choice of nonpharmacological treatment is balanced between patient demographics and the medical opinion of the clinician as there are several modalities (Figure 1) [33]. There is a demand for additional studies to specify the manner and type of nonpharmacological treatment along with their progression, intensity, and duration according to individualized patient outcomes [34].

Pharmacological treatment

The choice of pharmacologic therapy is predominantly dependent on chronic pain requiring the differentiation of nociceptive pain from neuropathic pain. The patient’s overall medical profile can affect the drug of choice due to drug interactions, clearance, and side effects [34]. If the patient does not improve from the nociceptive treatment, then reconsider if the patient has nociplastic or neuropathic pain and modify the management plan [34].

Nonsteroidal anti-inflammatory drugs (NSAIDs), in vivo, act directly on spinal nociceptive processing with a relative order of potency which corresponds with their capacity as cyclooxygenase activity inhibitors and centrally mediated analgesics, making them the drug of choice for musculoskeletal pain [35,36]. However, they are associated with a slight significant improvement in disability and chronic low back pain [35]. As per a study published in 2015, topical NSAIDs provide pain relief in acute conditions like strains, overuse injuries, and sprains, particularly gel formulations of ibuprofen, ketoprofen, and diclofenac with minimal adverse effects [37]. In a 2010 prospective, randomized, unblinded pilot study, 20 patients with chronic knee pain received either ibuprofen three times daily or 4% topical gel four times daily for two weeks. The study found that topical ibuprofen provided the same clinical efficacy as oral ibuprofen (Table 3) [38]. According to the World Health Organization, the recommended first-line treatment in all pain-related conditions is acetaminophen [39]. In a comparison review regarding the efficacy of ibuprofen and acetaminophen in chronic and acute pain conditions, a more significant number of patients reported a higher degree of pain relief when treated with ibuprofen [40]. This study also highlighted the equal importance of both medications as it also found that both drugs are needed as neither drug will be more effective for many patients [40]. In a randomized controlled trial in Hong Kong, 300 adult patients with musculoskeletal lower limb pain were given acetaminophen, NSAID, and acetaminophen-diclofenac combination therapy (Table 3) [41]. There was an insignificant difference in the average reduced intensity of the pain score between any treatment. All the combinations proved safe, and the acetaminophen-diclofenac combination attained a lower pain score first (Table 3) [41].

There is a strong recommendation for using tricyclic antidepressants, serotonin- noradrenaline reuptake inhibitors, pregabalin, and gabapentin as a first-line treatment in neuropathic pain [42]. According to a meta-analysis conducted in 2015 including 229 studies, it was found that for one patient to have at least 50% pain relief, 3.6 to 6.4 patients with neuropathic pain needed to be treated with tetracycline antidepressants (TCAs) or serotonin-norepinephrine reuptake inhibitors (SNRIs) [42]. Specifically, chronic back discomfort is generally treated with antidepressant medication to reduce pain intensity and re-establish function [43]. A meta-analysis with 504 patients with chronic back pain included articles with randomized placebo-controlled trials of antidepressants were included. The analysis found that antidepressants are more effective in lowering pain severity, however not in the functional status of the patient [43]. Duloxetine, a selective serotonin-norepinephrine reuptake inhibitor, is already marketed to treat diabetic neuropathy, depression, and fibromyalgia [44]. In a randomized, double-blind, placebo-controlled trial published in 2010, 530 outpatients 18 years of age or older were randomized to duloxetine or placebo for a 24-week double-blind treatment (Table 3) [45]. This study found that patients treated with 60, 90, 120 mg/day duloxetine was associated with pain reduction, fewer sleep difficulties and less fatigue, stiffening, and improved functioning and mood (Table 3) [45]. Venlafaxine is another antidepressant that is used to reduce neuropathic pain [46]. In another randomized controlled trial conducted at Dicle University Medical Faculty, 60 types 2 diabetes mellitus outpatients with painful peripheral diabetic neuropathy were randomly given venlafaxine. Those in the control group received a combination of vitamin B1 and B6 tablets (Table 3) [47]. The study concluded that venlafaxine lowered the severity of pain, and was a well tolerable analgesic drug (Table 3) [47].

Antiepileptic drugs like pregabalin and gabapentin have been used to manage several types of neuropathic pain and even fibromyalgia (Table 3) [48]. According to a study performed in 2013, gabapentin and pregabalin were helpful in painful diabetic and postherpetic neuralgia, with pregabalin being more effective in central neuropathic pain and fibromyalgia (Table 3) [48]. The above study’s findings can be compared in parallel to another study performed in 2017, where gabapentin at doses of 1800 mg to 3600 mg can show good levels of pain relief to some patients with peripheral diabetic neuropathy and postherpetic neuralgia [49]. pregabalin seems to have efficacy in the management of central pain [50]. Although it relieves anxiety and disturbed sleep in individuals, some common side effects are ataxia, weight gain, dizziness, and drowsiness [50]. It is recommended for patients who take multiple drugs; however, there may be additive central nervous system-related side effects [50]. In a double-blind placebo-controlled trial published in 2004, 238 patients with postherpetic neuralgia were randomized to receive pregabalin of 150 mg/day or 300 mg/day or placebo for 81 weeks with the purpose to assess the efficacy and safety of said drug (Table 3) [51]. The drug’s effectiveness was observed within the first week along with the frequent adverse effects discussed above including drug mouth and headache as well (Table 3) [51]. A population-based cohort study in Sweden followed 191, 973 individuals from 2006 till 2013 who collected prescriptions for gabapentinoids (gabapentin or pregabalin) (Table 3) [52]. The aim of this study was to explore the adverse outcomes related to the drugs taken, and 5.2% of participants were treated for suicidal behavior or died from suicide, 36.7% presented with bodily injuries, 4.1% were arrested due to violent crimes, and 6.3% had a road traffic incident (Table 3) [52]. Therefore, this study proved that gabapentinoids should be used with caution as they are associated with an increased risk of coordination disturbances, criminal acts, and mental health issues (Table 3) [52].

**Table 3:** Summary of the studies showing outcomes of various pharmacological treatment modalities NSAIDs- Nonsteroidal anti-inflammatory drugs

Reference	Study Type	Population	Conclusion
Tiso et al. 2010 [38]	A Prospective, randomized, unblinded pilot study	20 patients	Management of chronic knee pain with topical ibuprofen was comparable with oral ibuprofen.
Woo et al. 2005 [41]	Randomized Controlled Trial	300 adult patients with painful isolated limb injuries	NSAIDs, paracetamol, and paracetamol-diclofenac combinations all proved to be equally safe.
Arnold et al. 2010 [45]	Randomized, double-blinded, placebo-controlled trial	A total of 530 outpatients, 18 years of age or older, participated. Patients must meet the American College of Rheumatology criteria for fibromyalgia	Management with duloxetine was associated with pain reduction, lowering sleeping difficulties, and overall improvement in mood and functioning.
Kadiroglu et al. 2008 [47]	Randomized controlled trial	60 type 2 diabetes mellitus	Venlafaxine is safe and well-tolerated in patients with painful peripheral diabetic neuropathy with minimal adverse effects.
Wiffen et al. 2013 [48]	Systemic Review	Cochrane Database. 3 tiers involving less than 200 participants each	Supported use of gabapentin and pregabalin in some neuropathic conditions and fibromyalgia.
Sabatowski et al. 2004 [51]	Randomized double-blind, placebo-controlled trial	238 patients with neuropathic pain from postherpetic neuralgia	Pregabalin treated neuropathic pain of postherpetic neuralgia along with improving sleep habits
Molero et al. 2019 [52]	Population-based cohort study	191,973 people from the Swedish Prescribed Drug Register	Gabapentinoids are linked with an increased risk of head/ body injuries, unintentional overdoses, and suicidal behavior

Opioids have always been one of the essential classes of medications that have been used in refractory cases of chronic pain [53]. In a meta-analysis of randomized trials of patients with chronic pain, the benefits of opioid use were statistically significant with low improvement to pain and disability and increased occurrences of vomiting [53]. Comparisons of opioids to nonopioid use suggested that the benefit for pain and functioning may be similar, although the data were from studies of low to moderate quality [53]. The Center for Disease and Control (CDC) recommends that opioids be only used when the benefits for function and pain are more significant than the risks [54]. Clinicians should prescribe the lowest dosage and reassess the risks and benefits before adjusting the dose [54]. The physician should re-evaluate the harms and benefits when the dosage has been increased to 50 morphine milligrams or more per day and they should reassess the patient's therapy every three months or less [54]. Although the CDC does not mention a limit on how long a patient can be on opioid therapy, the long-term use is related to fatal overdose, opioid use disorder, and myocardial infarctions [54]. For opioid therapy to continue, the benefits must always outweigh the risks [54]. There is a wide range of pharmacological modalities tailored to each patient’s health state and the type of chronic pain syndrome [55]. Furthermore, there are a few limitations, such as the adverse effects of opioids that several patients cannot tolerate [55].

**Figure 2:**
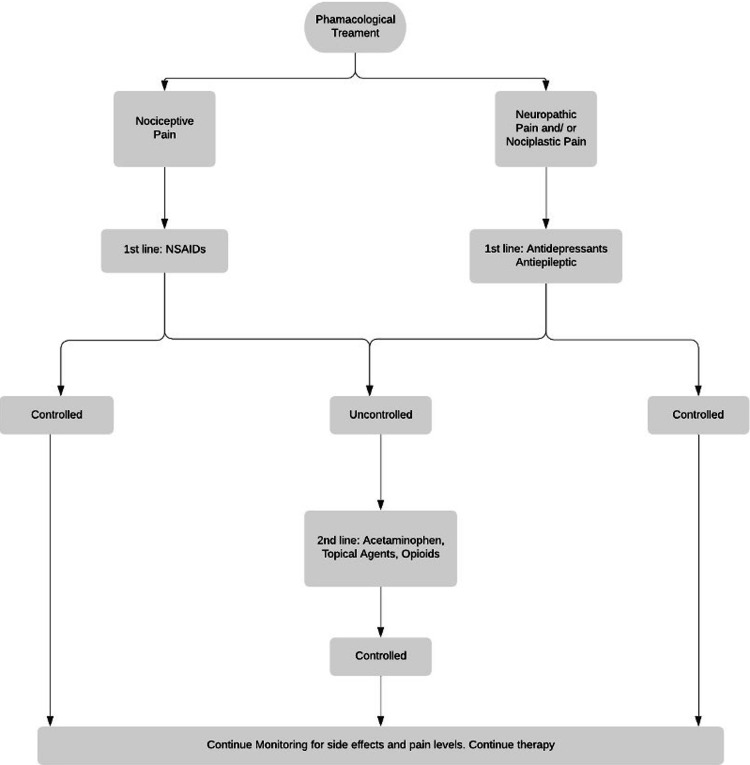
Algorithm for the pharmacological treatment of chronic pain NSAIDs- Nonsteroidal anti-inflammatory drugs

The first-line pharmacological management of neuropathic and nociplastic pain is usually a combination of antiepileptics and antidepressants, as the patients usually do not respond to a single agent (Figure 2) [42,56]. Whereas in cases of nociceptive pain, the treatment is typically guided by NSAIDs with various formulations and routes of administrations depending upon the location and character of pain [35,37]. Despite the treatment guidelines and the various data that prove the effectiveness of a range of pharmacological management (Table 3), the concept of chronic pain requires the management to be engineered according to the patients’ medical profile, type, and severity of chronic pain.

## Conclusions

As evident from the studies discussed in this article, nonpharmacological and pharmacological modalities play an equally important part in managing chronic pain. In summary, the clinical implication of this review article is to discuss the types of chronic pain and the various treatment options that are tailored to specific types of chronic pain such as physical therapy, psychological therapy, antidepressants, antiepileptics, etc. As evident from the discussion, chronic pain is a multifactorial condition requiring a comprehensive management plan. We believe that this article can serve as a guide to understand better the treatment options available and the preferred general algorithm when treating patients with chronic pain. We discussed the efficacy of multiple drugs and their degree of pain relief for specific types of chronic pain, along with multicentric management. Additionally, the article discussed the benefits of physical exercise and cognitive-based therapy and how it can help with the adverse effects of chronic pain. Furthermore, a better understanding of the type of chronic pain and the negative effects that the patient has will help to create an appropriate management plan, including nonpharmacological treatment along with pharmacological treatment, if required. However, we assume that there needs to be more in-depth research regarding physical modalities of nonpharmacological treatment such as the TENS as there is a lack of studies regarding the tolerance factor in the application of such a device and further exploration into the pathogenesis of nociplastic pain.
